# LPA-primed astrocytes induce axonal outgrowth of cortical progenitors by activating PKA signaling pathways and modulating extracellular matrix proteins

**DOI:** 10.3389/fncel.2014.00296

**Published:** 2014-09-25

**Authors:** Tania Cristina Leite de Sampaio e Spohr, Rômulo Sperduto Dezonne, Stevens Kastrup Rehen, Flávia Carvalho Alcantara Gomes

**Affiliations:** Instituto de Ciências Biomédicas, Universidade Federal do Rio de JaneiroRio de Janeiro, RJ, Brazil

**Keywords:** extracellular matrix, lysophospholipid, neuron–glia interaction, PKA pathway, lysophosphatidic acid

## Abstract

Lysophosphatidic acid (LPA) is one of the main membrane-derived lysophospholipids, inducing diverse cellular responses like cell proliferation, cell death inhibition, and cytoskeletal rearrangement, and thus is important in many biological processes. In the central nervous system (CNS), post-mitotic neurons release LPA extracellularly whereas astrocytes do not. Astrocytes play a key role in brain development and pathology, producing various cytokines, chemokines, growth factors, and extracellular matrix (ECM) components that act as molecular coordinators of neuron–glia communication. However, many molecular mechanisms underlying these events remain unclear—in particular, how the multifaceted interplay between the signaling pathways regulated by lysophospholipids is integrated in the complex nature of the CNS. Previously we showed that LPA-primed astrocytes induce neuronal commitment by activating LPA1–LPA2 receptors. Further, we revealed that these events were mediated by modulation and organization of laminin levels by astrocytes, through the induction of the epidermal growth factor receptor (EGFR) signaling pathway and the activation of the mitogen-activated protein (MAP) kinase (MAPK) cascade in response to LPA (Spohr et al., 2008, 2011). In the present work, we aimed to answer whether LPA affects astrocytic production and rearrangement of fibronectin, and to investigate the mechanisms involved in neuronal differentiation and maturation of cortical neurons induced by LPA-primed astrocytes. We show that PKA activation is required for LPA-primed astrocytes to induce neurite outgrowth and neuronal maturation and to rearrange and enhance the production of fibronectin and laminin. We propose a potential mechanism by which neurons and astrocytes communicate, as well as how such interactions drive cellular events such as neurite outgrowth, cell fate commitment, and maturation.

## Introduction

Lysophospholipids (LPs) are not only structural components of cellular membranes but also biologically active molecules. LPs were initially identified as precursors and metabolites of de novo biosynthesis of phospholipids. However, it was subsequently observed that they have properties similar to those of extracellular growth factors or signaling molecules, influencing processes such as carcinogenesis, neurogenesis, immunity, vascular development, or regulation of metabolic diseases. Lysophospholipids act through specific G protein-coupled receptors in an autocrine or paracrine fashion (for review see Ishii et al., 2004; Grzelczyk and Gendaszewska-Darmach, 2013).

Lysophosphatidic acid (LPA) is one of the main membrane-derived LPs. Lysophosphatidic acid signaling properties are mediated by at least six G protein-coupled receptors, referred to as LPAR1–LPAR5 and P2Y5 (Gardell et al., 2006; Choi et al., 2010). Lysophosphatidic acid-induced cellular responses include cell proliferation, cell death inhibition, and cytoskeletal rearrangement, which play important roles in many biological processes such as oncogenesis, wound healing, immune functions, and especially brain development (Anliker and Chun, 2004; Choi et al., 2010). Lysophosphatidic acid receptors are expressed in subsets of cells in the developing and mature rodent nervous system and play key functions in its morphogenesis and homeostasis (Choi et al., 2010). In the central nervous system (CNS), post-mitotic neurons release LPA extracellularly (Fukushima et al., 2000) whereas astrocytes do not (Spohr et al., 2008).

Astrocytes, the most abundant glial cells, play a key role in brain development and pathology, producing various cytokines, chemokines, growth factors, and extracellular matrix (ECM) components which act as molecular coordinators of neuron–glia communication (Yuan et al., 1997; Sloan and Barres, 2014). Moreover, they have been shown to be potential stem cells in the developing or in the adult brain (Kriegstein and Alvarez-Buylla, 2009), to influence neuronal fate and growth-cone navigation (Wislet-Gendebien et al., 2005; Spohr et al., 2008; Dezonne et al., 2013), to modify the number of synaptic connections (Ishige et al., 2001), and to control synapse formation and function (Shi and Massague, 2003; Corbin et al., 2008; Allen et al., 2012; Diniz et al., 2012). Because astrocytes express all five LPA classic receptors (LPA1–5; Spohr et al., 2008; Noguchi et al., 2009), they are potential targets for LPA actions on neuronal tissue.

Much is known about neuron–glia interactions and their role in CNS development, neuronal differentiation, and axonal outgrowth (De Bock et al., 2014; Sloan and Barres, 2014). However, many molecular mechanisms underlying these events remain unclear—in particular, how the multifaceted interplay between the signaling pathways regulated by lysophospholipids is integrated in the complex nature of the CNS.

We previously described how astrocytes treated with LPA induce neuronal differentiation and neurite outgrowth of cortical neurons. These events are mediated by a soluble factor secreted by astrocytes and able to induce the epidermal growth factor (EGF) signaling pathway, activate mitogen-activated protein (MAP) kinase (MAPK), and rearrange the astrocyte laminin ECM (Spohr et al., 2008, 2011). Indeed, it is well known that ECM proteins, such as laminin and fibronectin, play a key role in neuronal differentiation and regeneration, neurite elongation, neuronal migration, proper axonal projection, and synaptogenesis (Carri et al., 1988; Chamak and Prochiantz, 1989; Nakashima et al., 1999; Nat et al., 2007).

It is still unknown whether LPA affects the production and rearrangement of fibronectin by astrocytes and if so, how this process affects neuronal differentiation and maturation of cortical neurons. In the present work we aimed to answer these questions, and accordingly investigated the mechanisms involved in these processes. Here, we report for the first time that LPA-primed astrocytes affect fibronectin levels and deposition by activating protein kinase A (PKA) signaling pathways in response to LPA. Our data provide new insights on the role of astrocytes as inductors of neuronal fate commitment and neurite outgrowth, associated with PKA pathway activation in treated astrocytes.

## Material and methods

### Astrocyte primary cultures

Animals were obtained from the Animal Facility of the Institute of Biomedical Sciences of the Federal University of Rio de Janeiro. All animal protocols were approved by the Animal Research Committee of the Federal University of Rio de Janeiro (DAHEICB 024). Astrocyte primary cultures were prepared from the cerebral cortex of newborn Swiss mice, as previously described (Spohr et al., 2008). Briefly, after the mice were anesthetized, they were decapitated, the brain structures were removed, and the meninges were carefully stripped off. Tissues were washed in phosphate-buffered saline (PBS) with 0.6% glucose (Sigma Chemical Co., St. Louis, MO, USA) and dissociated into single cells in a medium consisting of Dulbecco’s minimum essential medium (DMEM) and nutrient mixture F12 (DMEM/F12; Invitrogen, Rockville, MD, USA), enriched with glucose (3.3 × 10^−2^ M), glutamine (2 × 10^−3^ M), and sodium bicarbonate (0.3 × 10^−2^ M). Dissociated cells were plated onto glass coverslips on a 24-well plate (Corning Incorporated, NY, USA), previously coated with poly-L-lysine (Sigma Chemical Co.), in DMEM/F12 medium supplemented with 10% fetal bovine serum (Invitrogen, Carlsbad, CA, USA). The cultures were incubated at 37°C in a humidified 5% CO_2_, 95% air chamber for 7–10 days until reaching confluence.

### LPA treatment and conditioned medium preparation

After reaching confluence, glial monolayers were washed three times with serum-free DMEM/F12 medium and incubated as previously described for an additional day in this medium. After this period, the cultures were treated with 1 μM LPA (Spohr et al., 2011) (Avanti Polar Lipids, Alabaster, AL, USA) in DMEM/F12 supplemented with 0.1% fatty-acid-free bovine serum albumin (FAFBSA, Sigma Chemical Co.) for 4 h. Control astrocyte carpets were treated with DMEM/F12 supplemented with 0.1% FAFBSA. Then, the glial monolayers were washed with serum-free DMEM/F12 medium, and the medium was replaced by DMEM/F12 without serum and used as the substrate in neural progenitor–astrocyte assays. For astrocyte conditioned medium preparation, after the astrocyte monolayers were treated with LPA-FAFBSA or FAFBSA, the medium was replaced by DMEM-F12 and the cultures were then maintained for an additional day. Conditioned medium (CM) derived from either LPA-treated astrocytes (LPA-CM) or conditioned medium of control astrocytes (C-CM) was recovered, centrifuged at 1500 g for 10 min, and used immediately or stored in aliquots at −70°C for further use.

### Neural progenitor–astrocyte co-culture assays

For cortical progenitor cultures, female Swiss mice were killed by ether followed by cervical dislocation, and embryos were removed at day 14 (E14). Cortical progenitors were prepared from the cerebral cortex of E14 embryos as previously described (Spohr et al., 2008). Briefly, cells were freshly dissociated from the cerebral cortex and 5 × 10^4^ cells were plated onto glial monolayer carpets that were either not treated or were previously treated with LPA for 4 h or 300 μL of LPA-CM, as previously described. In the case of LPA-CM assays, the medium was not replaced after 4 h of treatment; instead, it was left until the end of the co-culture. The co-cultures were kept for 24 h at 37°C in a humidified 5% CO_2_, 95% air atmosphere.

### Neural progenitor cultures

Progenitor cells were prepared from cortex derived from 14-day Swiss mice embryos, as described above. Briefly, cells were freshly dissociated from the cerebral cortex, and 1 × 10^5^ cells were plated onto glass coverslips previously coated with poly-L-lysine (Sigma Chemical Co.) in astrocyte-conditioned medium. The cultures were kept for 24 h at 37°C in a humidified 5% CO_2_, 95% air atmosphere (Spohr et al., 2011).

### PKA inhibition assays

For PKA inhibition assays, the PKA inhibitor KT5720 was used. Astrocyte monolayers were concomitantly treated with LPA (1 μM) and specific signaling pathway inhibitors KT5720 (400 nM) for 4 h according to the previously described protocol. After the treatment, embryonic precursors (E14) were plated onto the treated astrocyte carpets and co-cultures were kept at 37°C in a humidified 5% CO_2_, 95% air atmosphere for 24 h. The inhibitor was purchased from Calbiochem (Gibbstown, NJ) and diluted in methyl sulfoxide (C_2_H_6_OS, Sigma Chemical Co., St. Louis, MO).

### Immunocytochemistry

Immunocytochemistry was performed as previously described (Spohr et al., 2011). Briefly, cultured cells were fixed with 4% paraformaldehyde for 30 min and permeabilized with 0.2% Triton X-100 for 5 min at 24°C. After permeabilization, the cells were blocked with 5% bovine serum albumin (BSA) (Sigma Chemical Co.) in PBS (blocking solution) for 1 h and incubated overnight at 4°C with specified primary antibodies diluted in blocking solution. Primary antibodies were mouse anti-β-tubulin III antibody (Promega Corporation, Madison, WI, USA; 1:1000), rabbit anti-fibronectin (Sigma Chemical Co., 1:100), and rabbit anti-laminin (Sigma Chemical Co., 1:100). After primary antibody incubation, cells were extensively washed with PBS and incubated with secondary antibodies for 1 h, at 24°C. The secondary antibodies were goat anti-mouse IgG conjugated with alexa fluor 488 (Molecular Probes, Eugene, OR, USA; 1:500) and goat anti-rabbit IgG conjugated with alexa fluor 546 (Molecular Probes, 1:500). Negative controls were performed by omitting the primary antibody during staining. In all cases, no reactivity was observed when the primary antibody was absent. Cell preparations were mounted directly on n-propyl gallate (Sigma Chemical Co.) and visualized using a Nikon microscope.

### Immunoblotting assays

Protein concentration in cell extracts (CE) was measured in triplicate with the BCA™ Protein Assay kit (Cole-Parmer Canada Inc., Montreal, QC, Canada). Fifty micrograms of protein per lane were electrophoretically-separated in 8–15% gradient (for ECM proteins) sodium dodecyl sulfate–polyacrylamide gel. After separation, the proteins were electrically transferred to a Hybond-P polyvinylidene difluoride (PVDF) transfer membrane (Amersham Biosciences, Little Chalfont, Buckinghamshire, UK) for 3 h. Membranes were blocked overnight in Tris-buffered saline-Tween 20 (Merck, Darmstadt, Germany) containing 10% BSA. Then, primary antibodies diluted in block solution were added for 2 h at 24°C. After several washes in Tris-buffered saline-Tween 20, peroxidase-conjugated secondary antibodies diluted in block solution were added to the membrane and incubated for 2 h at 24°C. Proteins were visualized using the enhancing chemiluminescence detection system (Super Signal West Pico Chemiluminescent Substrate; Pierce Biotechnology, Milwaukee, WI, USA), and polyvinylidene difluoride membranes were exposed to autoradiographic films. Primary antibodies were mouse anti-α-tubulin (Sigma Chemical Co., 1:5000), rabbit anti-fibronectin (Sigma Chemical Co., 1:500) and rabbit anti-laminin (Sigma Chemical Co., 1:500). Secondary peroxidase-conjugated antibodies were goat anti-rabbit IgG and goat anti-mouse IgG (Amersham Biosciences, 1:5000).

### Quantitative analysis

To determine cell density, neuron number, and neurite outgrowth, the co-cultures were immunostained with the antibody against the neuronal marker β-tubulin III (Promega Corporation, 1:1000) and visualized and counted using a Nikon microscope. Neurite length was assessed by measuring the distance from one cell body to the end of all neurites, in which the final length was considered the sum of all neurites measured from this one cell body. We also measured the longest neurite by measuring only the distance from one cell body to the end of the longest neurite. At least 10 fields were measured per well. In all cases, at least 100 randomly chosen neurons were observed per well (Spohr et al., 2011). The experiments were done in triplicate, and each result represents the mean of three independent experiments. Statistical analyses included a Student’s *t*-test and a one-way ANOVA, followed by a Tukey’s Multiple Comparison Test. Densitometry of the immunocytochemistry images and the blotting gels was performed using the Image J 1.40 g program (National Institutes of Health, USA).

## Results

### LPA-primed astrocytes induce fibronectin rearrangement

**Figure 1:**
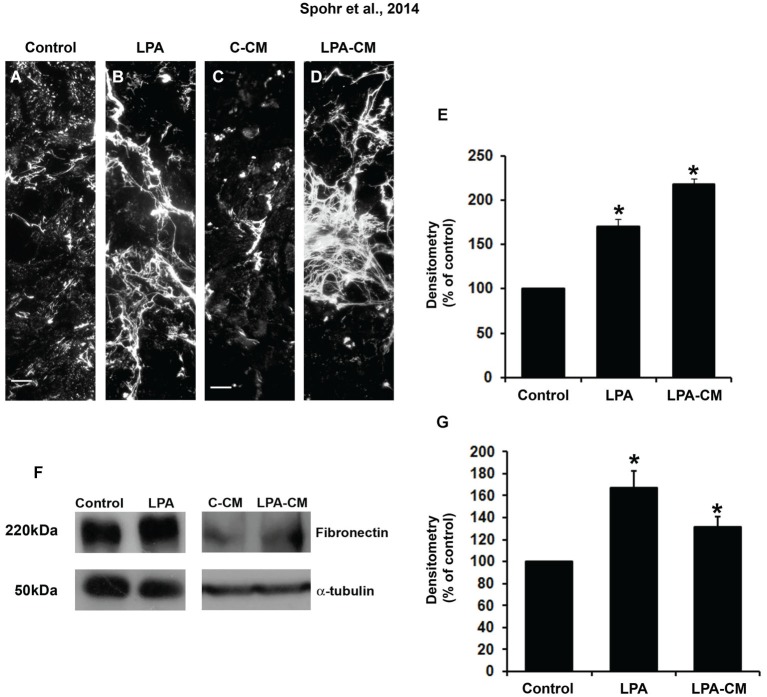
**LPA-primed astrocytes modulate astrocyte levels of ECM proteins *in vitro***. After confluence, astrocytes were treated for 4 h with FAF-BSA **(A)** or 1 μM LPA **(B)**, or control conditioned medium (C-CM) **(C)**, or conditioned medium derived from LPA-treated astrocytes (LPA-CM) **(D)** for 24 h. Then the cells were fixed and immunostained for the ECM protein fibronectin **(A–D)**. We observed an increase in the levels and a difference in the deposition pattern of fibronectin expressed by the astrocytes when they were treated with LPA and LPA-CM **(A–E)**. The levels of fibronectin per micrograph were analyzed in ImageJ (percentage of control). Representative Western blots and graphic analysis were done from three independent experiments. After treatment of astrocytes, equal amounts of total protein (50 μg per lane) from astrocytes treated with LPA **(F)** were analyzed by immunoblotting for fibronectin. Immune reaction for α-tubulin was used to monitor the loading. The levels of fibronectin protein immunoreactivity are expressed relative to the levels observed in cultures of astrocytes treated with FAF-BSA (control astrocytes). For **(E, G)**, * *p* < 0.05. Error bar corresponds to SEM. All quantifications were made at least three times. Scale bars correspond to 20 μm.

To investigate whether fibronectin ECM was modulated by astrocytes, we treated it with LPA for 4 h or with LPA-CM for 24 h. After 24 h, these cultures were fixed and immunostained for fibronectin (Figure 1). Both LPA and LPA-CM significantly affected fibronectin rearrangement compared to control conditions (Figures 1A–D). In addition, LPA- or LPA-CM-treated astrocytes increased fibronectin protein by 70% and 118%, respectively, as measured by immunolabeling densitometry (Figure 1E) or by 67% and 17%, respectively, as measured by Western blot (Figures 1F,G). These data suggest that LPA modulates fibronectin rearrangement by stimulating astrocytes through direct or indirect mechanisms.

### LPA-primed astrocytes induce neuronal differentiation and maturation by PKA pathway activation

The signaling pathways of LPA receptors recruit different G proteins, which in turn activate various downstream effectors such as Rho, Ras/MAPkinase, and phospholipase C (Panetti et al., 2001). Thus, the response to LPA is determined by which pathway is activated. Cyclic AMP (cAMP) controls many cellular processes, mainly through the activation of PKA, such as integrin-mediated cell adhesion (Ribatti, 2007).

**Figure 2:**
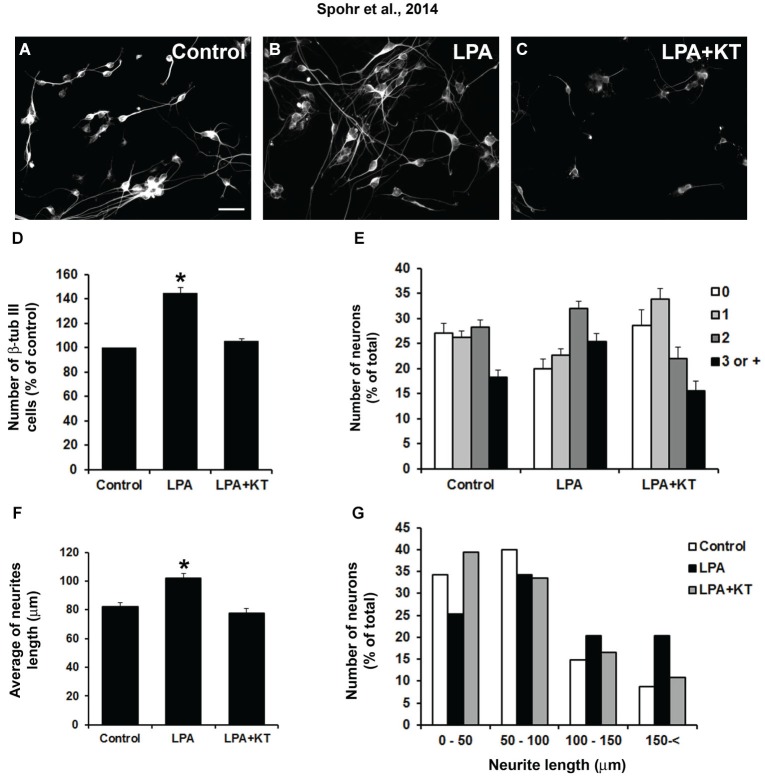
**LPA-treated astrocytes induce neuronal differentiation through PKA signaling pathway**. Cortical progenitors obtained from E14 mice were cultured for 24 h on control astrocytes **(A)** or LPA-treated astrocytes alone **(B)** and simultaneously with PKA inhibitor KT5720 (LPA + KT; **C)**. Cells were fixed and immunostained for the neuronal marker β-tubulin III (β tub III). Addition of PKA-specific inhibitor totally abolished the effect of LPA on neuronal differentiation **(D)**, arborization **(E)** and neurite outgrowth **(F and G)**. Statistical significance was observed for all columns (0, 1, 2, 3, or + neurites) between control–LPA and LPA–LPA + KT (*p* < 0.05). Total neurite length **(F and G)** was obtained using the ImageJ Software (National Institutes of Health, USA). In all cases, at least 100 randomly chosen neurons were observed. All quantifications were made at least 3 times. For **(D)** and **(F)**, * *p* < 0.05; error bars correspond to SEM. Scale bar corresponds to 30 μm.

We questioned whether PKA is involved in neurite outgrowth and is induced in LPA-primed astrocytes. To this end, astrocyte monolayers were treated for 4 h concomitantly with LPA and the specific PKA inhibitor KT5720. To prevent a direct action of the drug on neurons rather than a glia-mediated effect, inhibitor-containing medium was replaced by drug-free medium before neuronal plating; after 24 h, the cells were stained for β-tubulin III (Figures 2A–C). After 24 h, we observed an increase of 44% in the neuronal population plated onto LPA-primed astrocytes (Figures 2B,D). In addition, morphological analysis revealed a marked increase in the number of processes per neuron plated onto LPA-primed astrocytes, with an overall increase of those neurons showing two or more neurites (Figure 2E). Cells were grouped according to the number of processes extending from a single soma. LPA-primed astrocytes decreased the number of aneuritic neurons relative to control cultures (Figure 2E). However, inhibition of PKA reverted LPA-induced neuronal differentiation, neuritogenesis, and neurite outgrowth to those observed in the control conditions (Figures 2D–F). Most neurons plated onto astrocytes treated with PKA inhibitor developed neurites with average size between 50 and 100 μm, which was similar to the neurons under control conditions. On the other hand, neurons plated onto LPA-primed astrocytes exhibited an average size between 100 and 200 μm (Figure 2G). Thus, PKA is required for LPA-primed astrocyte-induced neuronal maturation.

### PKA pathway is necessary in LPA-primed astrocytes for fibronectin and laminin protein deposition and rearrangement

**Figure 3:**
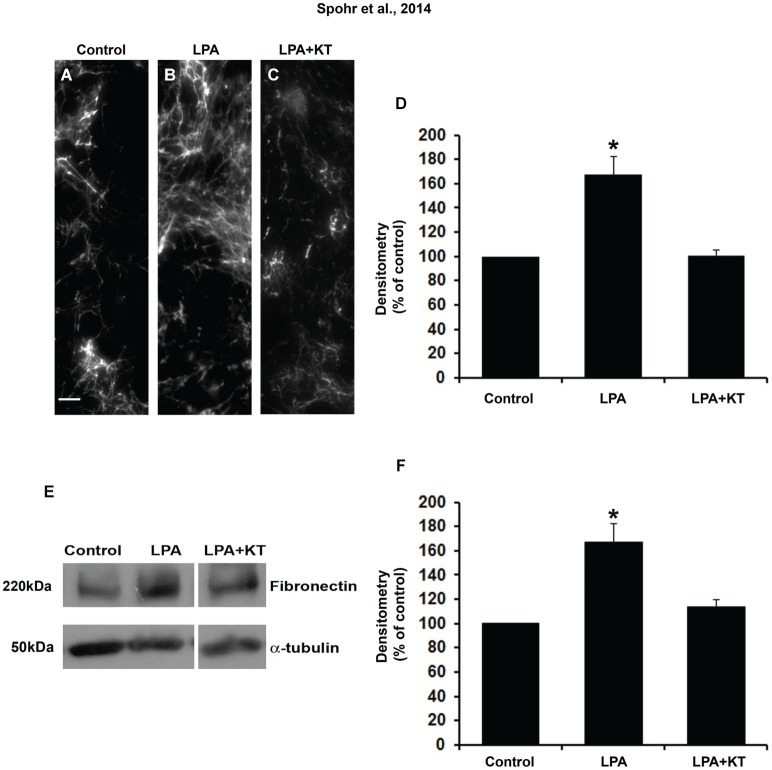
**PKA specific inhibitor totally abolished the effect of LPA on fibronectin levels**. After confluence, astrocytes were treated for 4 h with FAF-BSA **(A)** or 1 μM LPA **(B)**, or simultaneously with PKA specific inhibitor KT5720 **(C)**. After 24 h, the cells were fixed and immunostained for FN **(A–C)**. KT5720 abolished the effect of LPA on FN protein levels, **(A–F)**. Representative Western blots and graphic analysis of three independent experiments showing FN protein levels **(E)**. Immune reaction for α-tubulin was used to monitor loading. KT5720 abolished LPA effects on FN levels (k). For **(D, F)**, *p < 0.05; error bars correspond to SEM. Scale bar corresponds to 20 μm.

Because LPA-primed astrocytes induce neuronal differentiation and maturation by PKA activation, we investigated if fibronectin deposition was associated with PKA activation. To evaluate this question, astrocyte monolayers were simultaneously treated with LPA and KT5720 for 4 h. Fibronectin rearrangement was analyzed by immunostaining and fibronectin production by Western blot (Figures 3A–F). Immunostaining densitometry results suggest that the PKA inhibitor KT5720 reverted LPA-induced fibronectin rearrangement to control levels (Figure 3D). Further, KT5720 also reduced LPA-induced total fibronectin production by astrocytes to the levels observed under control conditions (Figure 3F).

**Figure 4:**
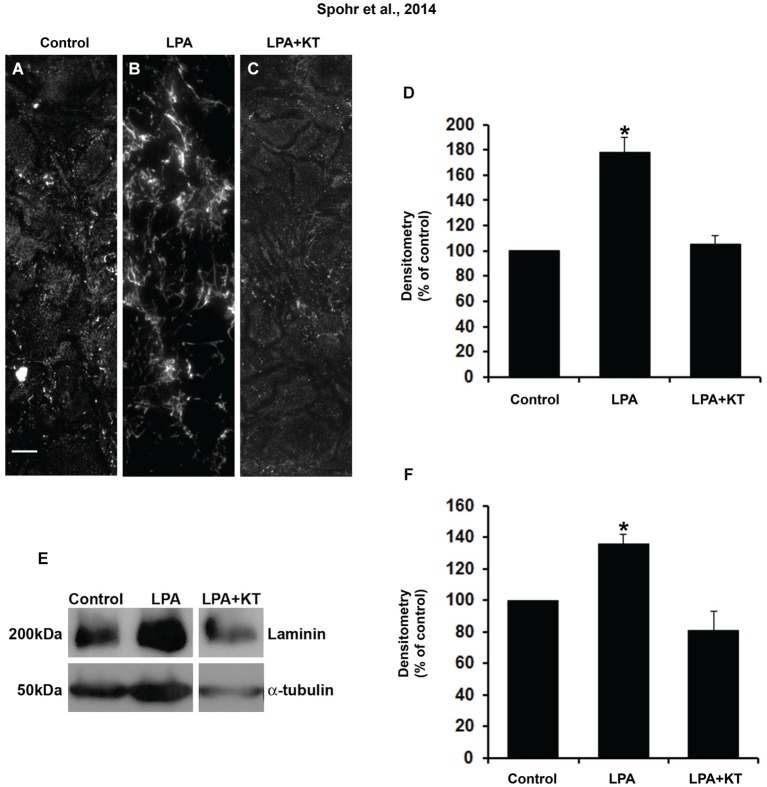
**PKA specific inhibitor totally abolished the effect of LPA on laminin levels**. After confluence, astrocytes were treated for 4 h with FAF-BSA **(A)** or 1 μM LPA **(B)**, or simultaneously with PKA specific inhibitor KT5720 **(C)**. After 24 h, the cells were fixed and immunostained for laminin **(A–C)**. KT5720 abolished the effect of LPA on LN protein levels **(A–D)**. Representative Western blots and graphic analysis of three independent experiments showing LN protein levels **(E)**. Immune reaction for α-tubulin was used to monitor loading. KT5720 abolished LPA effects on LN levels **(F)**. For **(D), (F)**, * *p* < 0.05; error bars correspond to SEM. Scale bar corresponds to 20 μm.

We also observed that PKA inhibition abolished laminin rearrangement and total production in LPA-primed astrocytes (Figures 4A–F). This was shown either by immunostaining densitometry (Figure 4D) or Western blot (Figure 4F). Thus, PKA activation is required for LPA-primed astrocytes to induce neurite outgrowth and neuronal maturation and to rearrange and enhance the production of fibronectin and laminin.

## Discussion

In the present work, we investigated whether LPA stimulates astrocytes to rearrange and produce fibronectin and, if so, how this process affects neuronal differentiation and maturation. Furthermore, we explored whether a similar mechanism is employed by laminin.

Our previous studies using LPA1–LPA2 *knockout* mice showed that LPA-primed astrocytes induce neuronal commitment by activating both of these receptors in astrocytes (Spohr et al., 2008). Further, we revealed that these events were mediated by modulation and organization of laminin levels by astrocytes, through the induction of the epidermal growth factor receptor (EGFR) signaling pathway and the activation of the MAPK cascade in response to LPA (Spohr et al., 2011). The results presented here suggest that the PKA signaling pathway and fibronectin are also important for LPA-primed astrocytes to induce neuronal maturation. Because the PKA pathway is activated, it is possible that the LPA receptor isoform LPAR4 is also involved in this process (Gardell et al., 2006).

Fibronectin and other ECM proteins have been shown to influence neurite outgrowth (Chamak and Prochiantz, 1989; Garcia-Abreu et al., 1995; Martinez and Gomes, 2002; Guizzetti et al., 2008; Plantman et al., 2008), axonal guidance (Webber et al., 2008), differentiation (Ma et al., 2008; Sun et al., 2008), and cell proliferation (Wang and Milner, 2006; Lathia et al., 2007).

It is already known that LPA enhances binding and modulates the assembly of fibronectin on the surface of non-neural cells (Zhang et al., 1994). This is the first report to show that LPA modulates fibronectin rearrangement and production in the nervous system. Because PKA inhibition in LPA-primed astrocytes also abolished the effects of neuronal commitment and neurite outgrowth, it is plausible that fibronectin plays a key role in this event. In a previous study from our group, we described that EGF induces neurite outgrowth of cerebellar neurons by modulating the content of laminin and fibronectin on the astrocyte surface, thus enhancing cerebellar neuritogenesis *in vitro* (Martinez and Gomes, 2002). However, in this study we were not able to establish a direct correlation between the two events in cortical astrocytes.

Fibronectin is an important ECM protein that participates in cellular adhesion, spreading, and migration of diverse cell types (Trentin et al., 2003), whereas laminin helps in neurite outgrowth as an adhesive substrate (Garcia-Abreu et al., 1995; Spohr et al., 2011). Modulation of these two ECM proteins is therefore important for normal CNS development. Other molecules such as guanosine 5′-monophosphate and guanosine have been shown to activate PKA and other signaling effectors such as MAPK, protein kinase II, Ca^2+^-calmodulin and protein kinase C, in order to reorganize ECM proteins (Decker et al., 2007). Here we also observed that PKA signaling is important for fibronectin and laminin production and rearrangement in LPA-primed astrocytes.

Interestingly, LPA is not the only lysophospholipid able to modulate ECM proteins and neuronal maturation. Recently we showed that shingosine-1 phosphate (S1P) increased and modified the pattern of astrocytic laminin production to promote neuronal differentiation of neural progenitor cells (Spohr et al., 2012). We observed that S1P-primed astrocytes also affect fibronectin rearrangement and production (data not shown).

LPA induces several cellular responses, depending on the cell type, the receptor subtype, and the effector pathway stimulated (Choi et al., 2010). In cultured astrocytes, LPA treatment induces intracellular Ca^2+^ mobilization, production of reactive oxygen species, and DNA synthesis, and also inhibits glutamate uptake (Tabuchi et al., 2000; Bergers and Benjamin, 2003; Spits et al., 2008). In addition, LPA treatment also induces morphological changes, stabilization of astrocyte stress fibers, and stimulates actomyosin contraction through a Rho-mediated pathway (Breier, 2000; Weaver et al., 2007). Further, PKA and RhoA activation are involved in cellular migration induced by LPA (Hirakawa et al., 2007). Also, LPA and αβ and β integrins act in concert to activate Rac1, RhoA, PKA, and PI3K to promote cell polarization and motility (O’Connor et al., 2012). Thus, besides PKA and MAPK, one can speculate that Rac1, RhoA, and Phosphoinositide 3-kinase also play a role in the downstream signaling events triggered by LPA, which culminate in astrocyte-induced neuronal maturation and fibronectin organization. However, further studies will be necessary to address this hypothesis.

**Figure 5:**
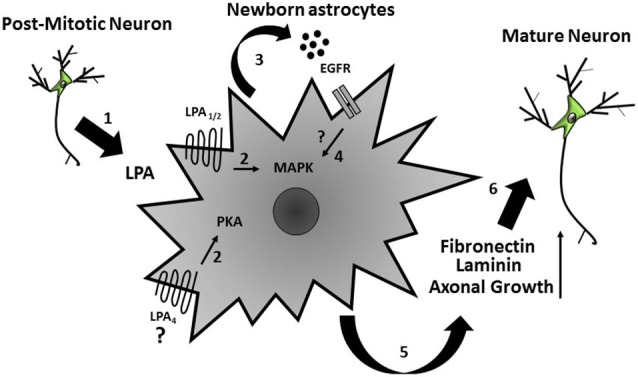
**Schematic model of the indirect effects on neural precursor cells mediated by LPA-primed astrocytes**. Extracellular LPA, possibly secreted by post-mitotic neurons (1), acts on cortical astrocytes and (2) induces secretion of EGF ligands (EGF and/or TGF-α) and activated MAPK and PKA signaling pathways in these cells (3) thus yielding production of ECM proteins (laminin and fibronectin) (4). These events lead to neuronal precursor differentiation and axonal growth (5).

Over the last decade, the neurosciences have gone through a paradigm change, due to accumulating evidence that glial cells, especially astrocytes, are key players in brain development and pathology (Gomes et al., 2001; De Bock et al., 2014; Sloan and Barres, 2014). Indeed, these cells may act as stem cells, either during development or in the adult brain (Kriegstein and Alvarez-Buylla, 2009), and control synapse formation and function (Diniz et al., 2012). Here we propose a potential mechanism by which neurons and astrocytes communicate, as well as how such interactions drive cellular events such as neurite outgrowth, cell fate commitment, and maturation. In summary, we suggest that post-mitotic neuronal extracellular LPA acts on astrocytes, which in turn secrete EGF ligands (EGF and/or TGF-α), activate MAPK and PKA pathways, and modulate fibronectin and laminin rearrangement and production (Spohr et al., 2011; Figure 5). These events are important to support neuronal precursor differentiation and neurite outgrowth mediated by LPA-primed astrocytes (Spohr et al., 2008, 2011). Future efforts should investigate the possibility of using this bioactive lipid as a therapeutic tool for axon regeneration and CNS repair.

## Conflict of interest statement

The authors declare that the research was conducted in the absence of any commercial or financial relationships that could be construed as a potential conflict of interest.

## Abbreviations

BSA: bovine serum albumin
C-CM: conditioned medium of control astrocytes
CNS: central nervous system
DMEM: Dulbecco’s modified Eagle’s medium
E 14: embryos at day 14
ECM: extracellular matrix proteins
EGF: epidermal growth factor
FAFBSA: fatty acid free bovine serum albumin
LPA: lysophosphatidic acid
LPA1–LPA5: the five LPA receptor subtypes
LPA-CM: conditioned medium of LPA-treated astrocytes
LPAR: lysophosphatidic acid receptors
MAPK: mitogen-activated protein (MAP) kinase
PBS: phosphate saline buffer
PKA: protein kinase A
PVDF: polyvinylidene difluoride
S1P: sphingosine 1-phospate
SDS-PAGE: sodium dodecyl sulfate-polyacrylamide gel
TBS-T: tris saline buffer with tween.
